# 泽布替尼治疗Bing-Neel综合征2例

**DOI:** 10.3760/cma.j.cn121090-20250907-00415

**Published:** 2026-05

**Authors:** 倩 王, 祎琳 孔, 蕾 曹, 姝超 秦, 建勇 李, 磊 范, 奕 夏, 祎 缪

**Affiliations:** 1 南京医科大学第一附属医院（江苏省人民医院）血液科，南京 210029 Department of Hematology, The First Affiliated Hospital of Nanjing Medical University, Nanjing 210029, China; 2 徐州市中心医院血液科，徐州 221000 Department of Hematology, Xuzhou Central Hospital, Xuzhou 221000, China

例1，男，64岁，患者2022年5月以乏力伴记忆力下降起病，2022年11月于当地医院查头颅MRI增强示脑膜弥漫性增厚、伴强化。PET-CT：腹膜后、双侧髂血管旁、骶前间隙、盆壁下增多软组织密度影，^18^F-氟代脱氧葡萄糖（FDG）代谢不均匀增高；右侧心膈角区、肝胃间隙、双侧髂外血管旁、腹股沟区多个淋巴结显示伴部分^18^F-FDG代谢轻度增高；脾大。免疫球蛋白（Ig）测定：IgM 28.8 g/L，血清免疫固定电泳：IgM-κ阳性。骨髓病理：髓腔内见大片B淋巴细胞浸润，符合淋巴瘤累及骨髓改变。骨髓细胞免疫分型：可见14.5％的异常小淋巴细胞。脑脊液（CSF）涂片见大量胞体中等或偏大的异常淋巴细胞。右侧腹股沟淋巴结活检病理：滤泡性淋巴瘤，Ⅰ级，滤泡区域>75％，见部分弥漫区域。结合患者临床表现及检查结果，考虑淋巴瘤累及中枢神经系统（CNS），予2个疗程的利妥昔单抗（RTX）+甲氨蝶呤（MTX）+脂质体多柔比星+环磷酰胺（CTX）+长春地辛+地塞米松方案化疗。2023年5月，患者因胆囊炎于我院（江苏省人民医院）行胆囊切除术，术后病理：（胆囊）慢性胆囊炎急性发作，胆石症，间质伴出血及纤维组织增生，灶性区肉芽组织形成，可见组织、泡沫细胞及多核巨细胞反应，含铁血黄素沉积，部分区域可见增生活跃的淋巴组织，考虑淋巴造血系统病变。2023年7月，患者意识障碍逐渐加重，出现意识不清伴呕吐，头颅CT示两侧基底节区、侧脑室旁腔隙性梗死及缺血灶。血常规示轻度贫血（HGB 95 g/L）；IgM 31.8 g/L；血清免疫固定电泳：IgM-κ阳性。骨髓细胞学及活检病理均符合B细胞慢性淋巴增殖性疾病（B-CLPD）侵犯骨髓表现，免疫分型：异常单克隆B淋巴细胞占有核细胞的6.3％，表达CD45、CD19、CD20dim、CD22、Kappa，不表达CD5、CD10、Lambda。并行腰椎穿刺+鞘内注射，CSF蛋白：1 259.7 mg/L。CSF流式细胞术：可见异常单克隆B淋巴细胞占有核细胞的36.7％。予大剂量MTX联合RTX+替莫唑胺方案化疗。患者出院后3 d，再次出现谵语、躁动、失眠。循环肿瘤DNA测序显示MYD88 L265P阳性。同时胆囊增生的淋巴组织免疫组化结果回报：结合苏木精-伊红（HE）染色观察情况、FISH检测结果（IGH/BCL2基因重排阴性）及基因检测结果（MYD88 L265P突变以及Ig重链/轻链基因重排克隆性检测结果均为阳性），判定为伴MYD88基因突变的B-CLPD。综上，患者IgM单克隆升高，MYD88 L265P突变阳性，骨髓检查符合B-CLPD，CNS侵犯，修正诊断为华氏巨球蛋白血症（WM）并发Bing-Neel综合征（BNS）。予RTX+泽布替尼+替莫唑胺方案治疗2个疗程，患者神志认知改善明显，此后口服泽布替尼长期维持治疗，截至2025年4月随访，状态良好。

例2，男，47岁，患者2022年8月以鼻塞伴淋巴结肿大起病，于当地医院查彩超示颈部双侧多发淋巴结肿大，遂至我院行颈部右侧淋巴结粗针穿刺活检，术后病理：符合惰性B细胞淋巴瘤。后患者未再进一步诊治。2023年5月，患者出现耳鸣、听力下降，于当地医院查血常规示中度贫血；IgM 4.9 g/L；免疫固定电泳：IgM-κ阳性。骨髓细胞学及活检病理均提示骨髓侵犯。免疫分型：小B细胞群占有核细胞的18.27％，主要表达CD19、CD10、HLA-DR、CD20、FMC7，Kappa阳性、Lambda阴性，不表达CD5、CD23、CD200、CD103、CD11c、CD25；浆细胞：CD38++，CD45弱阳性，SSC信号中等强度，同时表达CD38、CD19。外周血细胞基因检测：MYD88基因突变阳性，未检测到CXCR4突变。综上，患者诊断为WM，予苯达莫司汀联合RTX方案化疗3次。2023年8月，患者自觉颈部淋巴结较前进行性增大，有新发肿大淋巴结，伴有头部左侧痛、左眼睑下垂，左侧耳鸣、听力下降。完善PET-CT示：淋巴瘤治疗后较起病时：颈部双侧、双侧锁骨区、双侧腋窝、腹膜后、腹主动脉旁、双侧髂血管旁及双侧腹股沟多发淋巴结，病灶较前增大、增多，部分融合成团，较大者位于颈部右侧，^18^F-FDG代谢增高（SUVmax：6.3）；双侧扁桃体见软组织增厚，^18^F-FDG代谢增高（SUVmax：5.0）。全身骨髓^18^F-FDG代谢轻度增高；结合病史，符合淋巴瘤表现，Lugano分期为Ⅳ期。患者至我院行右侧颌下淋巴结活检，术后病理：结合HE染色镜下观察情况及既往病史，本例符合淋巴浆细胞性淋巴瘤；MYD88 L265P突变阳性。头颅MRI示双侧海绵窦、两侧框内球后结构、左侧颞部硬脑膜及左侧小脑幕异常强化，考虑淋巴瘤浸润可能，两侧视神经鞘膜积液。IgM 29.1 g/L；CSF蛋白930.5 mg/L，CSF脱落细胞学：镜下可见淋巴瘤细胞。CSF流式细胞术结果与骨髓免疫分型相同，CSF MYD88 L265P突变阳性。综上，患者确诊为BNS，予ZR-CHOP方案（泽布替尼+RTX+CTX+表柔比星+长春新碱+地塞米松）化疗2个疗程，后予泽布替尼联合RTX+大剂量MTX化疗2个疗程。患者症状较前改善，4个疗程后评估疗效达部分缓解。后续再予ZR-CHOP方案化疗4个疗程，期间共行腰椎穿刺+鞘内注射化疗药物［地塞米松+阿糖胞苷（Ara-C）+MTX］6次。8个疗程后评估疗效达非常好的部分缓解。2024年3月起，予ZR方案维持治疗（泽布替尼160 mg每日2次+RTX 600 mg每2个月1次）。2024年11月，复查CSF蛋白：715.4 mg/L。CSF脱落细胞学：可见成熟淋巴细胞，易见涂抹细胞。CSF流式细胞术结果显示：异常单克隆B淋巴细胞占有核细胞的8.0％，表达CD19、CD45、CD10、CD22、CD20、Kappa，不表达CD5、Lambda。骨髓细胞学：增生活跃，淋巴细胞比例增高。骨髓免疫分型：异常单克隆B淋巴细胞占有核细胞的0.038％。考虑患者病情进展，予泽布替尼联合大剂量MTX+Ara-C方案化疗3个疗程后，复查头颅增强MRI：左侧海绵窦区软组织增厚伴轻度强化，较前明显好转；垂体稍饱满，较前好转；脑内少许腔隙性脑梗死。CSF蛋白水平较前下降（567.1 mg/L），CSF脱落细胞学：全片未见有核细胞，提示疗效尚可，再予1个疗程泽布替尼联合大剂量MTX+Ara-C方案化疗。患者疾病恶性程度高，为进一步提高疗效，下一步治疗拟行自体造血干细胞移植，目前已完成自体造血干细胞采集。

